# Dichotomisation using a distributional approach when the outcome is skewed

**DOI:** 10.1186/s12874-015-0028-8

**Published:** 2015-04-24

**Authors:** Odile Sauzet, Mercy Ofuya, Janet L Peacock

**Affiliations:** Epidemiology and International Public Health, School of Public Health, Universität Bielefeld, Bielefeld, Germany; Division of Health and Social Care Research King’s College London and NIHR Biomedical Research Centre at Guy’s and St Thomas’ NHS Foundation Trust and King’s College London, London, UK

**Keywords:** Dichotomisation, Distributional method, Birthweight, High blood pressure, BMI, Gestational age

## Abstract

**Background:**

Dichotomisation of continuous outcomes has been rightly criticised by statisticians because of the loss of information incurred. However to communicate a comparison of risks, dichotomised outcomes may be necessary. Peacock et al. developed a distributional approach to the dichotomisation of normally distributed outcomes allowing the presentation of a comparison of proportions with a measure of precision which reflects the comparison of means. Many common health outcomes are skewed so that the distributional method for the dichotomisation of continuous outcomes may not apply.

**Methods:**

We present a methodology to obtain dichotomised outcomes for skewed variables illustrated with data from several observational studies. We also report the results of a simulation study which tests the robustness of the method to deviation from normality and assess the validity of the newly developed method.

**Results:**

The review showed that the pattern of dichotomisation was varying between outcomes. Birthweight, Blood pressure and BMI can either be transformed to normal so that normal distributional estimates for a comparison of proportions can be obtained or better, the skew-normal method can be used. For gestational age, no satisfactory transformation is available and only the skew-normal method is reliable. The normal distributional method is reliable also when there are small deviations from normality.

**Conclusions:**

The distributional method with its applicability for common skewed data allows researchers to provide both continuous and dichotomised estimates without losing information or precision. This will have the effect of providing a practical understanding of the difference in means in terms of proportions.

## Background

Researchers and practitioners in medicine often use continuous measurements to classify subjects as either normal or abnormal according to a particular cut-off. This dichotomisation is typically done for one of three reasons. The first is to facilitate a treatment decision for an individual, such as to give anti-hypertensive drugs if systolic blood pressure is over 160 mmHg. Secondly dichotomisation may be used to enable the quantification of the proportion in a population with abnormal outcome, such as the proportion of babies with low birthweight, i.e. birthweight under 2500 g. Thirdly dichotomisation is used to provide estimates that are more clinically meaningful for example in comparing two groups when a difference in say, mean birthweight in two groups may be difficult to interpret while a difference in the proportion with low birthweight is intuitively more meaningful. Dichotomisation is thus commonly seen and used but is known to be problematic because of the obvious loss of information and reduced statistical power.

The distributional approach [1,2] was developed to remedy this problem by providing a way to dichotomise a continuous outcome without losing precision by considering the proportion below a cut-off as a function of the mean and standard deviation of the distribution. In this way researchers may present both a mean difference and a comparison of proportions below a given cut-off with equivalent precision. With dual outcomes, the dichotomisation of continuous data is statistically rigorous.

The distributional method requires that the data follow a normal distribution or can be transformed to normal, for example by using a logarithmic transform. Many common health outcomes, e.g. blood pressure, body mass index (BMI), are not normally distributed because of perturbations due to the presence in the population of a few people with very high blood pressures or BMIs. This process has been described to lead to a skew-normal distribution of outcomes [3].

A small systematic review was undertaken to illustrate the ways in which three common outcomes, blood pressure, body mass index, and gestational age areanalysed and presented in medical journals. To do this the Pubmed database was searched using the terms blood pressure, body mass index (BMI), and gestationalage OR preterm birth and all their related Mesh terms. One hundred and ninety studies were retrieved, and after screening the full texts, 49 eligible studies wereidentified (blood pressure (BP): 23, BMI: 13, gestational age (GA): 13). Among the BP studies, analysis used the continuous data in 17/23 studies, dichotomous in 9/23 and both in 3/23. BMI was analysed as continuous in 9/13 studies and dichotomous in 5/13. One study included both continuous and dichotomous outcomes. Thepattern for GA was different as most studies (12/13) used the dichotomous form, while 3/13 used thecontinuous outcome and two studies use both forms. Over all three outcomes, authors rarely (4/49) commented on thedistribution of the data. Those are typical outcome for which the distributional method for dichotomisation could be beneficial because the population at risk are defined by a threshold. It is not known how robust the distributional method is to small deviations from normality. In this paper we investigate if the distributional method remains reliable in the case of deviations from normality and propose a generalisation of the distributional method to allow for skewness in distributions using the skew-normaldistribution.

## Methods

The methods section consists of two parts. In the first part we derive the estimates and standard error for the skew-normal distributional method for dichotomisation, and in the second part we provide the methods for two studies. The first study consists in illustrating the skew-normal method with real data and the second in assessing the robustness of the normal method to smalldeviation trom normality and to validate the skew-normal method through simulation. The research reported does not require any ethical approval due to its methodological nature.

### Distributional method for the dichotomisation of skewed data

#### The skew-normal distributional method

The normal distributional method has been previously described in detail [1] and [2]. In brief it provides a large sample approximation for the estimation of proportions and their standard errors assuming a normal distribution for the underlying population with parameters obtained from the data. The skew-normal distributional method uses the skew-normal distribution which has beenextensively studied in [3]. This distribution is a generalisation of the normal distribution which works by adding a third parameter *α* which defines the skewness (if *α*=0, the distribution is normal). The method of derivation of the distributional standard error for the proportion above or below a threshold is similar to one in [1] using the delta method.

Lets $\overline {X}_{n}$ be the sample mean of *n* independent identically skew-normal distributed random variables *X*_*i*_, *i*=1…*n* with mean *μ*, variance *σ*^2^ and skewness parameter *α*. Lets *x*_0_ be a threshold of interest. The random variable $p(\overline {X}_{n})$ for the proportion of the population with outcome value under the threshold *x*_0_ is defined as

(1) $$ {\small{\begin{aligned} p(\overline{X}_{n})=\int_{-\infty}^{x_{0}}2\frac{e^{\frac{-1}{2w^{2}}(t-(\overline{X}_{n}+\alpha'))^{2}}}{\sqrt{2\pi w^{2}}}\left(\int_{-\infty}^{\alpha(t-(\overline{X}_{n}+\alpha'))/w}\frac{e^{\frac{-1}{2}r^{2}}}{\sqrt{2\pi}}dr\right)dt  \end{aligned}}}  $$

where *α*^′^=*μ*−*wμ*_*z*_ and $w^{2}=\sigma ^{2}/(1-{\mu _{z}^{2}})$ with $ {\mu _{z}^{2}}=\frac {2}{\pi }\frac {\alpha ^{2}}{1+\alpha ^{2}}$ (see [3])

From the delta method we obtain that $ p(\overline {X}_{n})$ is approximately normally distributed with standard deviation

$$\frac{w^{2}}{\sqrt{n}}\left(1-{\mu_{z}^{2}}\right)p'(\mu)^{2} $$

We outline the derivation of $p'(\overline {X}_{n})$ the formula for the standard deviation in the Appendix.

Let *n*_1_, *n*_2_, *μ*_1_, *μ*_2_,*α*,*sd*, *p*_1_, and *p*_2_ be the sample sizes, the sample means, the pooled sample skew coefficient, the pooled sample standard deviation and the skew-normal distributional estimates of the proportions under the threshold *x*_0_ in each group for the two groups being compared. For each *i*=1,2, *αi*′=*μ*_*i*_−*w*_*i*_*μ*_*z*_.

Let *d*, *rr* and *or* be the skew-normal distributional estimates of the difference in proportions, risk ratio and odds ratio. The following formulae provide the variances (*se*^2^) for these estimates or theirlogarithm.

$$ {\fontsize{8}{12}{\begin{aligned} se(d)^{2}&\,=\,\!\frac{{w_{1}^{2}}}{\sqrt{n_{1}}}\left(1\,-\,{\mu_{z}^{2}}\right)\left(\!\frac{2e^{\frac{-1}{2{w_{1}^{2}}}(x_{0}-(\mu_{1}+\alpha_{1}'))^{2}}}{\sqrt{2\pi {w_{1}^{2}}}} \Phi(\alpha\frac{x_{0}-(\mu_{1}\,-\,\alpha_{1}')}{w_{1}}\right)^{2} \\ &+\frac{{w_{2}^{2}}}{\sqrt{n_{2}}}\left(1\,-\,{\mu_{z}^{2}}\right)\left(\!\frac{2e^{\frac{-1}{2{w_{2}^{2}}}(x_{0}-(\mu_{2}+\alpha_{2}'))^{2}}}{\sqrt{2\pi {w_{2}^{2}}}}\Phi(\alpha\frac{x_{0}\,-\,(\mu_{2}\,-\,\alpha_{2}')}{w_{2}} \right)^{2}\\ se(log(rr))^{2}&\,=\,\frac{1}{{p_{1}^{2}}}\!\frac{{w_{1}^{2}}}{\sqrt{n_{1}}}\left(1\!-{\!\mu_{z}^{2}}\right) \left(\!\!\frac{2e^{\frac{-1}{2{w_{1}^{2}}}(x_{0}-(\mu_{1}\!+\alpha_{1}'))^{2}}}{\sqrt{2\pi {w_{1}^{2}}}} \Phi(\alpha\frac{x_{0}\,-\,(\mu_{1}\!\,-\,\alpha_{1}')}{w_{1}}\!\!\right)^{2}\\ &+\!\frac{1}{{p_{2}^{2}}}\!\frac{{w_{2}^{2}}}{\sqrt{n_{2}}}\!\left(1\,-\,{\mu_{z}^{2}}\right)\!\left(\!\!\frac{2e^{\frac{-1}{2{w_{2}^{2}}}(x_{0}-(\mu_{2}+\alpha_{2}'))^{2}}}{\sqrt{2\pi {w_{2}^{2}}}}\Phi(\alpha\frac{x_{0}\,-\,(\mu_{2}\!\,-\,\alpha_{2}')}{w_{2}} \!\right)^{2}\\ se(log(or))^{2}&=\frac{1}{(p_{1}(1-p_{1}))^{2}}\frac{{w_{1}^{2}}}{\sqrt{n_{1}}}\left(1-{\mu_{z}^{2}}\right) \\&\quad\times \left(\frac{2e^{\frac{-1}{2{w_{1}^{2}}}(x_{0}-(\mu_{1}+\alpha_{1}'))^{2}}}{\sqrt{2\pi {w_{1}^{2}}}} \Phi(\alpha\frac{x_{0}-(\mu_{1}-\alpha_{1}')}{w_{1}}\right)^{2}\\ &+\frac{1}{(p_{2}(1-p_{2}))^{2}}\frac{{w_{2}^{2}}}{\sqrt{n_{2}}}\left(1-{\mu_{z}^{2}}\right)\\& \quad\times\left(\frac{2e^{\frac{-1}{2{w_{2}^{2}}}(x_{0}-(\mu_{2}+\alpha_{2}'))^{2}}}{\sqrt{2\pi {w_{2}^{2}}}} \Phi(\alpha\frac{x_{0}-(\mu_{2}-\alpha_{2}')}{w_{2}} \right)^{2} \end{aligned}}} $$

These standard errors use more information than the standard errors used for proportion estimate obtained from the data. They depend on the underlying distribution and not just on the sample proportion and sample size.

#### Proportions and transformed data

Transformed data presents difficulties of interpretation because it may not be possible to back-transform to the natural scale and even when this can be done, the meaning is changed. However the proportion below a cut-point is not affected if the transformation function is continuous and monotonic such as logarithm, square root, reciprocal etc. The proportions of patients with a condition defined by a threshold remain unchanged under a transformation of the outcome. In mathematical terms:

If *y* is an outcome and *Y* a certain threshold such that for example, if the outcome for patient *i*, *y*_*i*_ is smaller than *Y* then patient *i* is to be treated then for *f* a continuous increasing function

$$\text{if}~~ y_{i}<Y~~ \text{then}~~ f(y_{i})<f(Y). $$

And for *g* a continuous decreasing function then

$$\text{if}~~ y_{i}<Y ~~\text{then}~~ g(y_{i})>g(Y). $$

Among the usual functions used for transforming data, the logarithm, the square root and the square (all three applied only to positive values) are increasing functions. The inverse function (1/*x*) for positive outcomes or taking the opposite value (-x) are decreasing functions therefore a proportion in the lower tail in the original scale will be in the upper tail in the transformed scale.

### Study 1: Examples from data from several observational studies

To illustrate the use of the distributional method for the dichotomisation of skewed outcomes, we present the analysis of skewed data using the skew-normal distributional method and compare the results with the normal distribution method for transformed data. The data come from two observational studies: Birthweight (BW), body-mass index (BMI) and gestational age (GA) are outcomes taken from the St George’s Birthweight Study [4] and systolic blood pressure (SBP) was measured on stroke patients included in the South London Stroke Register[5,6] which was set up in 1995 and records all first-ever strokes in an inner city area of South London.

#### Study 2: Robustness to small deviation from normality and validation of the skew-normal method

We assess the robustness of the (normal) distributional method in the presence of skewness for two reasons: to find out if the results remain reliable even if the data are not exactly normally distributed and to establish the necessity of an alternative method for the case of data with more skewness. We also validate the the skew-normal method. Data were generated from 1. a lognormal distribution with skewed upper tails and 2. using a left and right skewed skew-normal distribution. The data were analysed using the normal distributional method and for the skew-normal data also using the skew-normal method. The log standard deviation $\sigma ^{2}_{\log }$ provides a measure of skewness for the lognormal data via the ratio of the expected value by the median which is equal to $\exp \left (\frac {\sigma ^{2}_{\log } }{2}\right)$. Values for the log standard deviation considered in this study range between 0.02 and 1. The parameter *α* of the skew-normal distribution was used as a measure of skewness for the skew-normal data ranging from -20 to 20. The values -1 and 1 provide small deviation from normality.

The validity of the distributional method is assessed through the bias of the estimate, how well the standard error (se) is an accurate measure of the variability of the estimate and the coverage of the 95% confidence interval of the true value. The varying parameters used for the simulation are the cut-point, the skewness (by varying the log standard deviation, from 0.02 to 1), the effect size (mean difference over standard error, from 0.01 to 0.5) and the sample size (20 to 500).

Simulations were performed using the statistical software *R*. The following algorithm was followed 20 000 times for each set of parameter values. For each simulated dataset, the mean and standard error are obtained to compute the normal distributional estimates with standard error for the difference in proportion, risk ratio and odds ratio.

Summaries are then obtained for the 20 000 datasets in the following way:

- Mean values over the 20 000 datasets are obtained for all estimates and standard errors.
- Standard deviations over the 20 000 datasets are also obtained for difference in proportions, RR and OR in order to be compared to the mean standard errors.
- The mean bias (defined as the relative difference between true values and estimates) is obtained for all estimates
- The coverage of the 95% distributional confidence interval (DCI) is computed as the proportion of datasets for which the true value of the parameter was in the DCI.

## Results

### Study 1: Skew-normal distributional method illustrated with data from several observational studies

#### Normal data

Data from the St George’s Birthweight study [4] were used to compare the proportions of low birthweight (LBW) babies among smoking and non-smoking mothers. Results are given in Table 1a.

**Figure 1:**
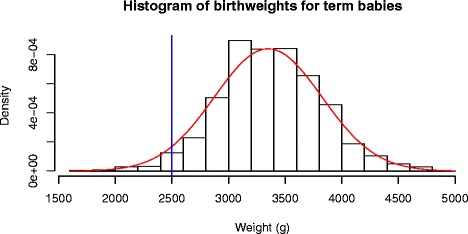
Histograms of birthweights for term babies with normal curve.

- Birthweight data for term babies is known to be normally distributed [7] (Figure 1) and the distributional method can be used without transformation.
- The mean BW (SD) in the non-smoking group was 3452g (435) for 983 observations and for the smoking group 3267g (441) for 494 observations
- The data are normally distributed (see above) and standard deviations can be assumed to be equal.
- The difference in means (SE) between smoking and non-smoking mothers is 184 (24) with 95% CI [137, 232]
- The normal distributional estimates for the difference in proportions in LBW between smoking and non-smoking mothers was 0.025 (0.004) with 95% DCI [0.017, 0.033].
- The skew- normal distributional estimates for the difference in proportions in LBW between smoking and non-smoking mothers was 0.024 (0.004) with 95% DCI [0.016, 0.032].

**Table 1:** **Application of the skew-normal method to some common outcomes and comparison with the normal method applied to transformed data**

**a. Proportions of low birthweight babies**						
N		Mean (SD)		Difference		
Non-smoker	Smoker	Non-smoker	Smoker	in means (SE)	95% conf. int.	*p*-value
983	494	3452g (435)	3267g (441)	184 (24)	[137, 232]	<0.001
Difference proportions		Risk ratio		Odds ratio		
Normal distributional estimates (no transformation)						
0.025 (0.004)	[0.017, 0.033]	2.68 (0.13)	[2.09, 3.43]	2.74 (0.13)	[2.13,3.54]	
Skew-normal distributional estimates						
0.024 (0.004)	[0.016,0.032]	2.87 (0.16)	[2.09,3.92]	2.94 (0.16)	[2.13,4.05]	
**b. Proportions of patients with high blood pressure**						
N		Mean (SD)		Difference		
Non-whites	Whites	Non-whites	Whites	in means (SE)	95% conf. int.	*p*-value
661	1235	149.2 (25.7)	144.1 (24.8)	5.11 (1.21)	[2.74, 7.49]	<0.001
Difference proportions		Risk ratio		Odds ratio		
Normal distributional estimates on the transformed scale						
0.068 (0.016)	[0.036,0.100]	1.28 (0.06)	[1.14,1.43]	1.40 (0.08)	[1.19,1.64]	
Skew-normal distributional estimates						
0.061 (0.017)	[0.028,0.093]	1.25 (0.06)	[1.11,1.40]	1.36 (0.08)	[1.15,1.60]	
**c. Proportions of obesity**						
N		Mean (SD)		Difference		
Primipari	Multipari	Primipari	Multipari	in means (SE)	95% conf. int.	*p*-value
891	890	22.96 (3.40)	23.84 (4.01)	0.88 (0.18)	[0.53,1.22]	<0.001
Difference proportions		Risk ratio		Odds ratio		
Normal distributional estimates on the transformed scale						
0.022 (0.005)	[0.013, 0.031]	1.66 (0.10)	[1.36, 2.02]	1.70 (0.11)	[1.38,2.09]	
Skew-normal distributional estimates						
0.020 (0.004)	[0.012,0.028]	1.40 (0.07)	[1.23,1.60]	1.44 (0.07)	[1.25,1.66]	
**d. Proportions of premature births**						
N		Mean (SD)		Difference		
Primipari	Multipari	Primipari	Multipari	in means (SE)	95% conf. int.	*p*-value
856	874	39.40 (1.96)	39.52 (2.12)	0.12 (0.10)	[-0.08, 0.31]	0.23
Difference proportions		Risk ratio		Odds ratio		
Normal distributional estimates on the transformed scale						
-0.019 (0.009)	[-0.037,-0.003]	0.82 (0.08)	[0.70,0.98]	0.81 (0.09)	[0.67,0.97]	
Skew-normal distributional estimates						
-0.010 (0.007)	[-0.024,0.006]	0.99 (0.06)	[0.97,1.01]	0.92 (0.07)	[0.80,1.05]	

DCI: distributional confidence interval.

#### Lognormal data

A dataset from The South London Stroke Registry provided the last recorded systolic blood pressure (SBP) before the first time stroke of 1896 patients. There are known differences in the risk of stroke for ethnic minorities in the UK[5,6] and here we look at the difference in proportions of high blood pressure between white and non-white patients. Results are given in Table 1b.

**Figure 2:**
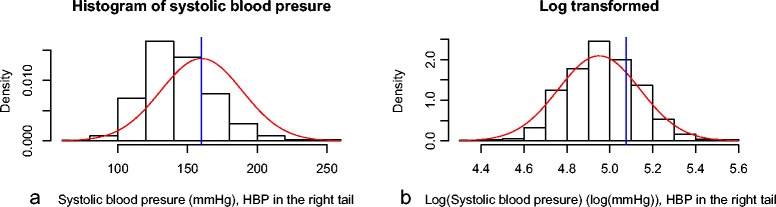
Histograms of systolic blood pressure (original and transformed scale).

- SBP is a right skewed outcome (see Figure 2a.) and the proportion of interest is in the right tail (patients with SBP ≥160). A logarithmic transformation provides a normally distributed outcome. In the transformed scale, high blood pressure patients are those with transformed SBP above log(160) =5.075.
- The mean (SD) SBP for the white ethnicity group was 144 mmHg (24) (transformed scale: 4.96 (0.17)) for 1235 observations and for the non-white group is 149 mmHg (26) (transformed scale: 4.99 (1.7)) for 661 observations.
- The transformed variable log(SBP) can be assumed to be normally distributed (see Figure 2b.) and the standard deviations to be equal.
- The mean difference in SBP is 5.11 (1.2) with 95% CI [2.74, 7.49] (original scale)
- The normal distributional method reflecting the difference means on the transformed scale provided estimates for the difference in proportions (SE) of high blood pressure between non-white and white patients of 0.068 (0.016) with 95% DCI [0.036, 0.100].
- The skew-normal distributional method reflecting the difference in means on the original scale provided estimates for the difference in proportions (SE) of high blood pressure between non-white and white patients of 0.061 (0.017) with 95% DCI [0.028, 0.093].

#### Inverse transformation

Data from the St George’s Birthweight study [4] were used to obtain the BMI from the height and weight of pregnant women at the beginning of pregnancy. The usual threshold of 30 kg/m ^2^ to compute the proportion of mothers with obesity was used. Results are given in Table 1c.

**Figure 3:**
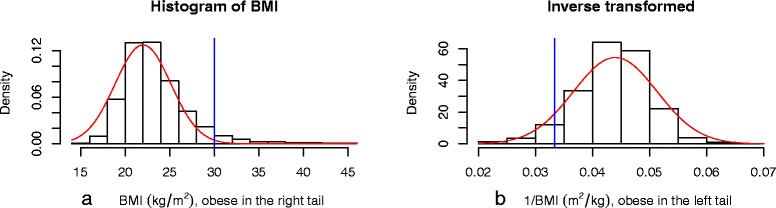
Histograms of BMI (original and transformed scales) with normal curve.

- The histogram of BMIs (Figure 3a.) showed a right skewed distribution. Taking the inverse of BMI provides a distribution which is approximately normal (Figure 3b.). We estimate the proportions of pregnant women with inverse BMI under 1/30 =0.033.
- The mean (SD) BMI in the multipari group was 23.8 (4.0) (transformed scale: 0.0430 (0.0062)) for 890 observations and for the primipari group was 23.0 (3.4) (transformed scale: 0.0444 (0.0059)) for 891 observations.
- The two groups can be assumed to have the same standard deviation.
- The mean difference in BMI between multipari and primipari was of 0.88 (0.16) with 95% CI [0.53, 1.22] (original scale).
- The normal distributional method reflecting the difference in means on the transformed scale provided estimates for the difference in proportions of 0.022 (0.005) with 95% DCI [0.013, 0.031].
- The skew-normal distributional method reflecting a difference in means on the original scale provided estimates for the difference in proportions of 0.020 (0.004) with 95% DCI [0.012, 0.029].

#### Other types of transformations

A newborn is considered preterm if its gestational age (GA) is under 37 completed weeks. Due to the natural termination and to medical intervention the duration of gestation does not normally go much over 43 weeks while there are a small number of very early birth, the distribution of GA is therefore left skewed.While we tried to perform a transformation, this one remains imperfect and the results show that using the skew-normal distributional method is the best alternative to reflect the difference means on the original scale. Results are presented in Table 1d.

**Figure 4:**
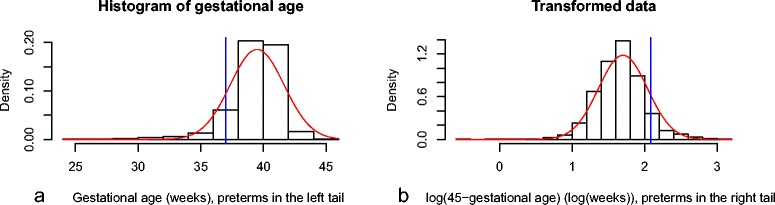
Histograms of gestational age (original scale and transformed) for term babies with normal curve.

- The first transformation is to take 45-GA which provides a right skewed positive outcome. Then a log transformation provides a fit close to normal (see Figure 4b.). We want to estimate the proportion of live births such that log(45-GA) >log(45-37)02.07 and
- There were 856 primipari mothers with mean GA of 38.34 weeks (1.96) (transformed scale: 1.64 (0.36)) and 874 multipari mothers with mean GA 39.52 weeks (2.12) (transformed scale: 1.67 (0.31)).
- The transformed data can be assumed to have a normal distribution and the standard deviations to be the same in both groups.
- The difference in means (SE) is 0.12 (0.10) with 95% CI [-0.08, 0.31] (original scale)
- The normal distributional estimate obtained on the transformed scale for the difference in proportions (SE) of pre-term live births between primipari and multipari mothers was 0.020 (0.009) with 95% DCI of [0.003, 0.037] (marginally significant reflecting a small significance for the mean difference in the transformed scale).
- The skew-normal distributional estimate obtained on the original scale for the difference in proportions (SE) of pre-term live births between primipari and multipari mothers reflecting the difference in means was 0.010 (0.007) with 95% DCI of [-0.024, 0.006].

### Study 2: Robustness of the distributional method and validation of the skew-normal method

Results of the simulations are summarised in Table 2 for the log-normal data and in Table 3 or the skew-normal data. Bias of estimates are summarised with the 3rd quantile of the absolute value. This shows that the bias for all sample size and skewness under 0.1 (log normal) remains small but then increases to level which may not be acceptable. For skew normal data, the normal method provides satisfactory results for small coefficients of skewness (±1 in these simulations). For RR and OR, the estimates are biased for small sample sized as seen in [2] but for sample size of 50 (100 for OR) per group or more the estimates are more robust to skewness that the difference in proportions. With increasing skewness the normal method is no more reliable but then the skew-normal method provides acceptable results for the skew-normal data. For small skewness parameter the skew normal method is unreliable and the normal method muss be used.

**Table 2:** **Summary of the simulation results per sample size per group and skewness (measured by the log-standard deviation)**

Bias of estimates*		**Summary statistic: 3rd quartile of the absolute value**							
**Log standard deviation**		**0.02**	**0.05**	**0.08**	**0.1**	**0.2**	**0.4**	**0.6**	**1**
	**Sample size**								
Diff. in prop.	20	0.03	0.03	0.05	0.06	0.08	0.38	0.94	2.20
	50	0.02	0.03	0.04	0.04	0.12	0.39	1.03	2.38
	100	0.01	0.03	0.03	0.05	0.11	0.41	1.04	2.46
	500	0.01	0.03	0.03	0.03	0.12	0.40	1.07	2.50
Risk ratio	20	0.09	0.09	0.09	0.09	0.07	0.05	0.07	0.10
	50	0.03	0.03	0.03	0.03	0.02	0.04	0.06	0.11
	100	0.02	0.01	0.01	0.01	0.02	0.03	0.06	0.10
	500	<0.01	<0.01	0.01	0.01	0.01	0.03	0.06	0.10
Odds ratio	20	0.24	0.25	0.25	0.25	0.25	0.22	0.23	0.32
	50	0.09	0.09	0.09	0.09	0.08	0.09	0.13	0.23
	100	0.04	0.04	0.04	0.04	0.04	0.06	0.11	0.21
	500	0.01	0.01	0.01	0.01	0.01	0.04	0.11	0.23
Bias of Standard errors**		**Summary statistic: 3rd quartile of the absolute value**							
**Log standard deviation**		**0.02**	**0.05**	**0.08**	**0.1**	**0.2**	**0.4**	**0.6**	**1**
	**Sample size**								
Diff. in prop.	20	0.02	0.02	0.03	0.03	0.03	0.03	0.04	0.06
	50	0.01	0.01	0.01	0.01	0.01	0.02	0.02	0.09
	100	0.01	0.01	0.01	0.01	0.01	0.02	0.02	0.10
	500	0.01	0.01	0.01	0.01	0.01	0.01	0.02	0.12
Risk ratio	20	0.04	0.05	0.05	0.05	0.04	0.04	0.04	0.07
	50	0.02	0.02	0.02	0.03	0.02	0.02	0.03	0.11
	100	0.01	0.01	0.01	0.01	0.02	0.02	0.03	0.11
	500	0.01	0.01	0.01	0.01	0.01	0.01	0.03	0.13
Odds ratio	20	0.04	0.05	0.05	0.05	0.05	0.04	0.03	0.04
	50	0.02	0.03	0.03	0.03	0.03	0.02	0.01	0.08
	100	0.02	0.02	0.02	0.02	0.03	0.02	0.01	0.08
	500	0.02	0.01	0.02	0.01	0.02	0.01	0.02	0.11
**Coverage of the 95% DCI**		**Summary statistic: Inter-quartile range**							
**Log standard deviation**		**0.02**	**0.05**	**0.08**	**0.1**	**0.2**	**0.4**	**0.6**	**1**
	**Sample size**								
Diff. in prop.	20	0.936	0.937	0.936	0.936	0.936	0.938	0.940	0.947
		0.946	0.944	0.945	0.947	0.948	0.950	0.951	0.956
	50	0.943	0.944	0.944	0.944	0.943	0.943	0.940	0.928
		0.950	0.948	0.949	0.949	0.949	0.950	0.949	0.955
	100	0.946	0.947	0.946	0.947	0.945	0.942	0.932	0.881
		0.950	0.950	0.950	0.950	0.950	0.948	0.947	0.944
	500	0.948	0.948	0.947	0.947	0.943	0.931	0.840	0.483
		0.951	0.950	0.950	0.950	0.950	0.945	0.935	0.834
Risk ratio	20	0.945	0.944	0.944	0.944	0.945	0.947	0.950	0.956
		0.957	0.956	0.955	0.956	0.959	0.962	0.965	0.972
	50	0.947	0.947	0.946	0.946	0.945	0.939	0.931	0.931
		0.954	0.953	0.952	0.952	0.954	0.954	0.954	0.962
	100	0.947	0.947	0.946	0.947	0.944	0.930	0.909	0.861
		0.951	0.951	0.951	0.952	0.951	0.951	0.953	0.950
	500	0.947	0.948	0.945	0.944	0.933	0.875	0.722	0.376
		0.951	0.950	0.950	0.951	0.950	0.948	0.925	0.850
Odds ratio	20	0.942	0.942	0.941	0.941	0.941	0.942	0.946	0.952
		0.945	0.945	0.945	0.945	0.946	0.946	0.950	0.960
	50	0.945	0.944	0.943	0.943	0.944	0.944	0.943	0.931
		0.949	0.948	0.948	0.948	0.948	0.949	0.951	0.958
	100	0.945	0.946	0.946	0.946	0.944	0.942	0.932	0.884
		0.949	0.949	0.949	0.949	0.949	0.949	0.950	0.945
	500	0.946	0.947	0.946	0.946	0.945	0.929	0.848	0.490
		0.950	0.950	0.950	0.950	0.949	0.948	0.932	0.833

*Mean of the relative difference between estimates and true parameter to the true paramter.

**Relative difference between the mean standard error and the standard deviation to the standard deviation.

Varying parameter include effect size (difference in mean) and cut-point.

**Table 3:** **Summary of the simulation results comparing skew normal and normal methods of dochotomisation per sample size per group and skewness of the skew-normal data**

Bias of estimates*		**Summary statistic: 3rd quartile of the absolute value**							
**Skewness (** ***α*** **)**		**±1**	**±5**	**±10**	**±20**	**±1**	**±5**	**±10**	**±20**
	**Sample size**	**Normal method**				**Skew normal method**			
Diff. in prop.	20	0.05	0.28	0.32	0.34	0.40	0.08	0.06	0.03
	50	0.05	0.31	0.34	0.34	0.37	0.02	0.02	0.02
	100	0.05	0.33	0.38	0.39	0.27	0.02	0.03	0.02
	500	0.04	0.33	0.38	0.40	0.09	0.01	0.01	0.01
Risk ratio	20	0.12	0.10	0.12	0.13	0.09	0.04	0.05	0.06
	50	0.02	0.11	0.13	0.12	0.10	0.03	0.03	0.03
	100	0.03	0.18	0.23	0.24	0.08	0.02	0.02	0.02
	500	0.03	0.20	0.23	0.24	0.02	0.01	0.01	0.01
Odds ratio	20	0.20	0.23	0.24	0.23	0.18	0.10	0.10	–
	50	0.09	0.07	0.20	0.25	0.25	0.05	0.0.05	0.05
	100	0.04	0.31	0.35	0.35	0.05	0.09	0.09	0.07
	500	0.01	0.32	0.35	0.34	0.01	0.01	0.02	0.01
Bias of Standard errors**		**Summary statistic: 3rd quartile of the absolute value**							
**Skewness (** ***α*** **)**		**±1**	**±5**	**±10**	**±20**	**±1**	**±5**	**±10**	**±20**
	**Sample size**	**Normal method**				**Skew normal method**			
Diff. in prop.	20	0.02	0.04	0.04	0.04	0.27	0.07	0.07	0.04
	50	0.01	0.03	0.03	0.03	0.34	0.02	0.02	0.02
	100	0.01	0.02	0.02	0.02	0.42	0.02	0.01	0.02
	500	0.01	0.01	0.02	0.02	0.45	0.01	0.02	0.02
Risk ratio	20	0.06	0.06	0.07	0.07	0.29	0.45	0.67	0.85
	50	0.03	0.05	0.05	0.05	0.36	0.05	0.04	0.05
	100	0.02	0.03	0.04	0.04	0.37	0.04	0.04	0.05
	500	0.02	0.02	0.03	0.02	0.38	0.03	0.02	0.03
Odds ratio	20	0.04	0.05	0.05	0.05	0.08	0.27	0.36	0.50
	50	0.02	0.03	0.03	0.03	0.03	0.02	0.01	0.08
	100	0.02	0.02	0.02	0.02	0.03	0.02	0.01	0.08
	500	0.02	0.01	0.02	0.01	0.10	0.03	0.02	0.03
**Coverage of the 95% DCI**		**Summary statistic: Inter-quartile range**							
**Skewness (** ***α*** **)**		**±1**	**±5**	**±10**	**±20**	**±1**	**±5**	**±10**	**±20**
	**Sample size**	**Normal method**				**Skew normal method**			
Diff. in prop.	20	0.935	0.918	0.915		0.914	0.917	0.916	0.917
		0.951	0.946	0.945	0.943	0.925	0.947	0.948	0.946
	50	0.943	0.918	0.912	0.907	0.711	0.942	0.937	0.939
		0.950	0.947	0.945	0.945	0.922	0.951	0.952	0.950
	100	0.944	0.889	0.873	0.869	0.720	0.944	0.942	0.942
		0.950	0.945	0.942	0.942	0.911	0.941	0.951	0.951
	500	0.943	0.617	0.571	0.535	0.842	0.948	0.947	0.945
		0.948	0.934	0.936	0.931	0.919	0.951	0.950	0.951
Risk ratio	20	0.942	0.922	0.918	0.918	0.819	0.932	0.934	0.934
		0.952	0.951	0.952	0.951	0.936	0.961	0.963	0.958
	50	0.945	0.900	0.893	0.888	0.778	0.942	0.942	0.940
		0.951	0.947	0.949	0.945	0.929	0.954	0.956	0.954
	100	0.941	0.763	0.709	0.706	0.759	0.943	0.944	0.941
		0.950	0.943	0.942	0.943	0.927	0.952	0.953	0.953
	500	0.947	0.948	0.945	0.944	0.867	0.945	0.945	0.943
		0.947	0.919	0.909	0.907	0.930	0.951	0.951	0.951
Odds ratio	20	0.944	0.931	0.925	0.926	0.938	0.941	0.939	0.939
		0.950	0.946	0.949	0.947	0.944	0.952	0.956	0.959
	50	0.944	0.890	0.888	0.884	0.938	0.944	0.945	0.944
		0.949	0.947	0.945	0.945	0.946	0.950	0.953	0.951
	100	0.942	0.787	0.759	0.768	0.931	0.944	0.945	0.944
		0.949	0.943	0.943	0.942	0.948	0.950	0.952	0.951
	500	0.933	0.293	0.216	0.222	0.932	0.944	0.945	0.944
		0.948	0.922	0.920	0.920	0.948	0.950	0.951	0.951

*Mean of the relative difference between estimates and true parameter to the true paramter.

**Relative difference between the mean standard error and the standard deviation to the standard deviation.

Varying parameter include effect size (difference in mean) and cut-point.

Bias of standard error defined as the difference between the mean standard error and the standard deviation relative to the standard deviation are summarised in Tables 2 and 3 with the 3rd quantile of the absolute value. This shows that the standard error reflects well the true variability of the parameter estimates unless the skewness is very large (log normal data) or if the sample size is small (20 per group) for the skew normal method.

The results for bias of estimates and of standard error are reflected in the coverage of the 95% (normal) distributional confidence interval also shown in Tables 2 and 3 with the interquartile range.

## Discussion

Our small review of the literature mentioned in the introduction showed that in 49 studies, only 4 authors described the distribution of their data. Skewed data were often analysed and presented as means, perhaps because they are easier to interpret on the original scale. Relatively few authors present both the continuous and dichotomous form of their outcome, when in fact the dual presentation provides a richer summary of the data. The distributional method provides a way to remedy this by providing dichomomised estimates that sits alongside its continuous outcome comparison but which does not lose power. However, the distributional method requires the data to follow a normal distribution and so we have sought to generalise the normal distributional method by adding a parameter and using the skew-normal distribution. We have performed two studies to complement the skew-normal method. In Study 2, we have seen using simulations that small deviation from normality did not affect the reliability of the normal distributional method, but for larger skewness a correction was required. We also saw that for larger skewness, the skew-normal method was reliable even for smaller sample sizes (50 per group or more, less so for 20 per group). In Study 1, we illustrated the skew-normal method with real data. We have shown with the gestational age example that a good transformation is not always available and the skew-normal distributional method is a better alternative. But more generally, the distributional method applied to transformed data will reflect the difference in means on the transformed scale (leading to potentially different conclusions) while both the skew-normal and normal distributional methods will reflect the difference in means in the original scale and the most appropriate should bepreferred.

In study 1, in the birthweight example we saw that for data almost normal the skew-normal and normal methods provided similar results. However the sample size in this dataset was large. Study 2 showed that for data almost normal the skew normal method did not perform well unless the sample size was large enough. The reason for this remains unclear but if the data looks normal and the sample size is nor large, the normal method should be preferred.

In this paper we presented only unadjusted estimates of comparison of proportions. But the method can be applied after a linear regression (also mixed models). Software are available [8] for Stata and *R*.

## Conclusion

This study has dealt with the two following issues: we have shown that the normal distributional method continued to perform well even if the actual distribution was slightly skewed showing that the method could be used with confidence with real data which will only be approximately normal. We have also generalised the method to include skewed data. The distributional method with its applicability for skewed data allows researchers to provide both continuous and dichotomised estimates without losing information or precision. This will have the effect of providing a practical understanding of the difference in means in terms of proportions.

## Appendix

We outline the derivation of $p'(\overline {X}_{n})$ of the skew-normal distributional proportion under the threshold *x*_0_. Formula 1 can be writen as the product of two functions:

$$p(\overline{X}_{n})=A(\overline{X}_{n})\times B(\overline{X}_{n}) $$

such that

$$\frac{d}{d\overline{X}_{n}} p(\overline{X}_{n})=A(\overline{X}_{n})\times \frac{d}{d\overline{X}_{n}} B(\overline{X}_{n})+\frac{d}{d\overline{X}_{n}}A(\overline{X}_{n})\times B(\overline{X}_{n}) $$

which gives

$$ \begin{aligned} \frac{d}{d\overline{X}_{n}}p(\overline{X}_{n})&=\int_{-\infty}^{x_{0}}(2/w^{2})(t-(\overline{X}_{n}+\alpha')) \frac{e^{\frac{-1}{2w^{2}}(t-(\overline{X}_{n}+\alpha'))^{2}}}{\sqrt{2\pi w^{2}}}\\ &\quad\times\left(\int_{-\infty}^{\alpha(t-(\overline{X}_{n}+\alpha'))/w}\frac{e^{\frac{-1}{2}r^{2}}}{\sqrt{2\pi}}dr\right)dt-\\ &\qquad\frac{2\alpha}{\sqrt{2\pi w^{2}}}\int_{-\infty}^{x_{0}}\frac{e^{\frac{-(\alpha^{2}+1)} {2w^{2}}(t-(\overline{X}_{n}+\alpha'))^{2}}}{\sqrt{2\pi w^{2}}}dt \end{aligned} $$

The first part can be simplified using an integration by parts giving

$$\begin{array}{*{20}l}  \frac{d}{d\overline{X}_{n}}p(\overline{X}_{n})&=-2\frac{e^{\frac{-1}{2w^{2}}(x_{0}-(\overline{X}_{n}+\alpha'))^{2}}}{\sqrt{2\pi w^{2}}} \Phi(\alpha(x_{0}-(\overline{X}_{n}-\alpha')/w)\\ &+2\frac{\alpha}{\sqrt{2\pi w^{2}}}\int_{-\infty}^{x_{0}}\frac{e^{\frac{-(1+\alpha^{2})}{2w^{2}}(t-(\overline{X}_{n}+\alpha'))^{2}}}{\sqrt{2\pi w^{2}}}-\\ &\qquad\frac{2\alpha}{\sqrt{2\pi w^{2}}}\int_{-\infty}^{x_{0}}\frac{e^{\frac{-(\alpha^{2}+1)} {2w^{2}}(t-(\overline{X}_{n}+\alpha'))^{2}}}{\sqrt{2\pi w^{2}}}dt \end{array} $$

The last two members of the equation simplifying, it remains that

$$p'(\overline{X}_{n})=-2\frac{e^{\frac{-1}{2w^{2}}(x_{0}-(\overline{X}_{n}+\alpha'))^{2}}}{\sqrt{2\pi w^{2}}} \Phi(\alpha(x_{0}-(\overline{X}_{n}-\alpha'))/w) $$

The value above is the building block to compute the standard error for the skew-normal distributional estimates of differences in proportions, risk ratios and odds ratios in a similar way as in [2] under the assumption of equal variance and equal skewness.
